# From Molecules to Machines: An Integrative Framework Linking Molecular Pathogenesis, Multi‐Factorial Risk, Risk Stratification, Clinical Management, and Artificial Intelligence in QT Prolongation and Sudden Cardiac Death

**DOI:** 10.1002/clc.70370

**Published:** 2026-06-11

**Authors:** Mojtaba Farjam, Mohammad Hosein Yazdanpanah, Narges Fereydouni

**Affiliations:** ^1^ Noncommunicable Diseases Research Center Fasa University of Medical Sciences Fasa Iran; ^2^ Center for Cardiac Arrhythmias, Massachusetts General Hospital Boston Massachusetts USA; ^3^ Clinical Research Development Unit, Valiasr Hospital Fasa University of Medical Sciences Fasa Iran

**Keywords:** artificial intelligence, long QT syndrome, metabolic risk factors, risk stratification, sudden cardiac death, torsades de pointes

## Abstract

QT prolongation causes torsades de pointes sudden death from heritable, pharmacologic, metabolic, nutritional triggers. Its dimensions have been studied separately. This integrative review synthesizes research on molecular pathogenesis, acquired/metabolic/nutritional risks, clinical stratification, therapy, and AI prediction. Dual‐function channel mutations and post‐translational defects underlie congenital LQTS beyond classic three genes. Drug–gene–metabolic interactions amplify acquired risk; insulin resistance, NAFLD, and adiposity are independent risk factors. Nutritional exposures (grapefruit juice, licorice, energy drinks) compound arrhythmic risk. QTc threshold alone is insufficient; T‐wave morphology, genotype, electromechanical window dynamics, and M‐FACT score add prognostic value. Nonpenetrant LQTS carries near‐population‐level event risk. Genotype‐targeted mexiletine and left cardiac sympathetic denervation are validated alternatives. Machine learning outperforms clinical scores; deep learning distinguishes congenital from acquired QT prolongation on ECG. Precision QT management requires integrated strategies including nutritional and metabolic determinants, QTc measurement, and AI‐enhanced prediction. Prospective data remain essential before algorithmic tools guide decisions.

## Graphical abstract

An integrative conceptual framework linking the five principal domains addressed in this review. Beginning at the molecular level, loss‐of‐function mutations in KCNQ1 and KCNH2 reduce outward repolarizing currents (IKs and IKr), while gain‐of‐function SCN5A mutations augment late inward sodium current (INa), establishing the genetic substrate for action potential prolongation and arrhythmogenesis. Acquired risk is superimposed through pharmacological QT prolongation, nonalcoholic fatty liver disease, and visceral adiposity—each acting as independent repolarization modifiers; nutritional risk factors including grapefruit juice, licorice, and energy drinks further amplify this risk through pharmacokinetic, electrolyte‐mediated, and gene–nutrient interaction pathways. Risk stratification extends beyond a single QTc threshold to incorporate genotype, T‐wave morphology, sex, symptomatic history, and the M‐FACT composite score, applied dynamically throughout the treatment course. Management follows a genotype‐informed escalation ladder from beta‐blockade through mexiletine, left cardiac sympathetic denervation (LCSD), and implantable cardioverter‐defibrillator (ICD) therapy. Artificial intelligence–based models, integrating ECG signal data with electronic health records and multimodal imaging, substantially outperform validated clinical risk scores for predicting drug‐induced QT prolongation and sudden cardiac death in retrospective cohort designs, with emerging capability to distinguish congenital from acquired QT prolongation at the ECG level. Bidirectional interactions across all five domains are illustrated: genetic substrate modifies risk expression from acquired and metabolic exposures; dynamic risk scoring drives and is updated by treatment response; and AI‐guided therapy selection depends on genotypic context for meaningful interpretation. Together, these five domains constitute a unified precision‐medicine framework for QT‐related arrhythmic risk. EHR, electronic health record; ICD, implantable cardioverter‐defibrillator; LCSD, left cardiac sympathetic denervation; NAFLD, nonalcoholic fatty liver disease.

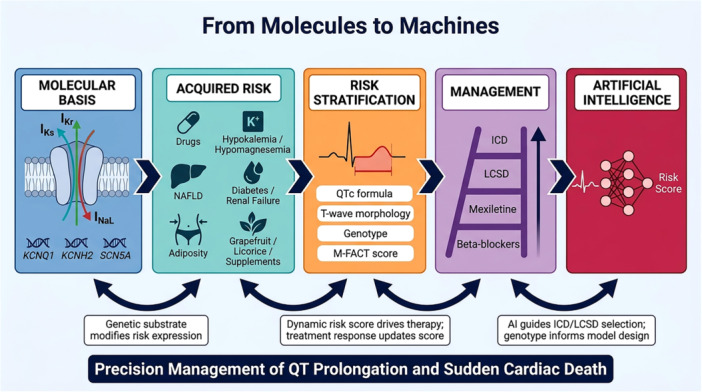

## 1 Introduction

Few conditions in clinical cardiology occupy as unusual a conceptual space as QT prolongation. It is at once a straightforward electrocardiographic measurement and a biological phenomenon of staggering complexity, linking nanoscale protein dynamics to population‐level mortality. The corrected QT interval (QTc) reflects the integrated repolarization behavior of hundreds of millions of ventricular cardiomyocytes, each dependent on the precisely orchestrated interplay of inward depolarizing and outward repolarizing ion currents. When that balance is disturbed—whether by germline mutation, pharmacological blockade, or metabolic derangement—the resulting repolarization heterogeneity creates the electrophysiological substrate for early afterdepolarizations and, ultimately, the potentially fatal polymorphic ventricular tachycardia known as torsades de pointes (TdP) [1, 2].

Sudden cardiac death (SCD) claims an estimated 350 000–450 000 lives annually in the United States alone [3], and a non‐trivial proportion of these events—particularly among the young and otherwise healthy—is attributable to conditions defined, at least in part, by abnormal ventricular repolarization [4, 5]. The congenital long QT syndromes (LQTS), caused by mutations in genes encoding cardiac ion channels and their regulatory proteins, collectively affect approximately 1 in 2000 individuals, though genuine population‐based penetrance estimates remain debated [2, 6, 7]. Drug‐induced LQTS is, in absolute terms, far more prevalent; it is the single most common reason that approved pharmaceuticals are withdrawn from the market or subjected to black‐box warnings, and it continues to complicate clinical decision‐making across intensive care, psychiatry, infectious disease, and gastroenterology practice [8, 9].

Despite decades of research, the management of QT prolongation has remained frustratingly empirical. Risk stratification has been dominated by the QTc threshold—a blunt instrument, as accumulating evidence makes clear—and therapeutic decisions have oscillated between undertreating high‐risk patients and overtreating low‐risk ones. The transformative potential of genotype‐guided therapy, dynamic risk reassessment, and precision pharmacology has been articulated in small but methodologically rigorous studies, yet implementation in routine clinical practice has lagged substantially. What has received considerably less attention, and what the emerging population‐based literature is beginning to make difficult to ignore, is the contribution of metabolic phenotypes—insulin resistance, hepatic steatosis, and regional adiposity—to the burden of acquired QT prolongation in the general population [10, 11, 12]. These metabolic risk factors are not merely incidental comorbidities; they appear to exert independent electrophysiological effects on ventricular repolarization through mechanisms that are only beginning to be characterized. Their systematic integration into QT risk frameworks is, at present, inadequate.

Against this background, the emergence of artificial intelligence (AI)‐based approaches, particularly deep learning models applied to the electrocardiogram (ECG), opens a genuinely new chapter in arrhythmic risk prediction—one that is only beginning to be interrogated at scale [13, 14]. At the same time, a foundational precondition for all QTc‐based risk assessment—the accuracy and population‐appropriateness of the correction formula used to derive QTc from the raw QT measurement—has received renewed empirical attention from population‐based cohort studies [15], a development whose implications for both clinical practice and AI model training deserve more acknowledgment than they have thus far received.

This review sets out to examine, synthesize, and critically evaluate the evidence base spanning five conceptually interdependent domains—molecular pathogenesis, acquired risk determinants, clinical risk stratification, therapeutic management, and artificial intelligence–based prediction—with the explicit aim of constructing an integrative analytical framework that the primary literature has, until now, largely failed to provide. The knowledge gap motivating this work is not the absence of evidence within any single domain; it is the structural fragmentation that has prevented mechanistic insights in one domain from informing clinical practice in another. Genotype‐specific therapeutic advances have not been systematically incorporated into risk stratification tools; metabolic risk determinants with established electrophysiological plausibility have been excluded from virtually all validated clinical instruments; and artificial intelligence models have been developed in almost complete isolation from the molecular and metabolic biology that governs the phenomena they claim to predict. The contribution of this review lies precisely in bridging these separations—offering a synthesis that traces the continuum from ion channel dysfunction through population‐level metabolic risk to algorithmic prediction, and that identifies, with empirical specificity, the points at which current clinical frameworks fail the patients they are designed to protect. The analysis is grounded in the most recent work published in leading journals in the field, prioritizing primary research of the highest methodological rigor available at this stage of the literature's development.

## 2 Methods

A structured literature search was conducted across PubMed, EMBASE, and Web of Science to identify the primary evidence base for this integrative review. The search was performed independently for each of the five conceptual domains addressed [1]: molecular and genetic pathogenesis of QT prolongation [2]; acquired, pharmacological, and metabolic risk determinants [3]; clinical risk stratification tools and their validation [4]; therapeutic management strategies; and [5] artificial intelligence–based arrhythmic risk prediction. For each domain, the chronological search proceeded from the most recent year (2026) backward to 2015, with mandatory prioritization of publications from 2025 to 2026. Where original primary research from 2025 or 2026 was identified and met inclusion criteria for a given domain, it was incorporated before older evidence was considered for that domain. Inclusion was restricted to original primary research articles—comprising experimental studies, prospective and retrospective cohort studies, randomized controlled trials, case‐control studies, and cross‐sectional studies with original data—published in high‐impact, peer‐reviewed journals and directly relevant to the specific domain under search. Review articles, systematic reviews, meta‐analyses, and narrative reviews were excluded as primary foundations but were used selectively to provide contextual grounding. Studies were evaluated for methodological rigor, population relevance, outcome validity, and verifiability of bibliographic details; studies from predatory, unrecognized, or regionally limited journals were excluded. The total primary evidence base was synthesized narratively across the five domains, with critical evaluation of study design, population characteristics, generalizability, and level of evidence at each analytical step.

## 3 Ion Channel Architecture and Molecular Pathogenesis of QT Prolongation

### 3.1 The Electrophysiological Logic of Repolarization

Ventricular repolarization—the process by which cardiomyocytes restore their resting membrane potential after each excitation—depends upon the coordinated activity of multiple ion channel species. The slow delayed rectifier current (IKs), encoded by the KCNQ1 gene in association with its β‐subunit KCNE1, and the rapid delayed rectifier current (IKr), generated by the hERG protein encoded by KCNH2, are the principal outward currents driving repolarization. The fast voltage‐gated sodium channel, Nav1.5 (encoded by SCN5A), carries the transient inward current responsible for depolarization, but its late component—a small residual inward current persisting well into phases 2 and 3 of the action potential—becomes pathologically amplified in certain genetic and pharmacological contexts, directly prolonging repolarization and predisposing to early afterdepolarizations (EADs) [2, 6, 16].

### 3.2 Genetic Architecture of Congenital LQTS

**FIGURE 1:**
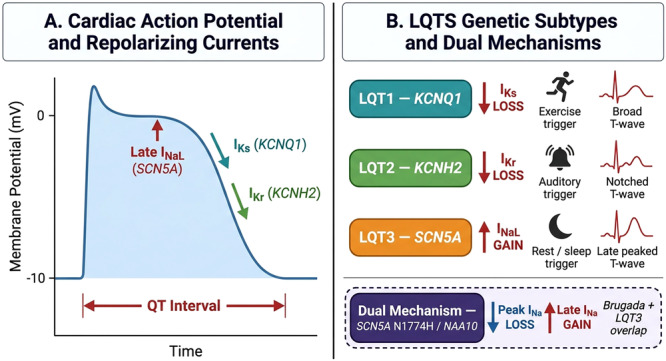
Ion channel molecular architecture and the genetic basis of congenital long QT syndrome. (A) The ventricular action potential illustrating the principal repolarizing currents (IKs and IKr) and the pathologically amplified late sodium current (INaL). (B) Genotype‐specific mechanisms, arrhythmic triggers, and characteristic T‐wave morphologies for LQT1, LQT2, and LQT3, together with the dual‐mechanism concept exemplified by variants producing simultaneous peak INa loss‐of‐function and late INa gain‐of‐function. LQTS = long QT syndrome.

Mutations in KCNQ1 (LQT1), KCNH2 (LQT2), and SCN5A (LQT3) together account for the vast majority of genotype‐positive congenital LQTS cases [6, 17]. The functional consequences of these mutations are mechanistically heterogeneous. In LQT1 and LQT2, loss‐of‐function variants reduce outward repolarizing current—through decreased channel expression, impaired trafficking, accelerated inactivation, or defective channel assembly—prolonging action potential duration and, by extension, the surface QT interval. In LQT3, gain‐of‐function mutations in SCN5A increase the late sodium current (INaL), adding an additional inward depolarizing burden during the repolarization phase. The principal repolarizing currents and their genotype‐specific dysfunction across the three major LQTS subtypes are illustrated in Figure 1.

This mechanistic dichotomy is not merely of academic interest; it directly governs genotype‐specific triggers and, more practically, informs therapeutic choices [18, 19]. LQT1 patients experience arrhythmic events predominantly during exercise or emotional stress, consistent with the established role of IKs upregulation in adrenergically mediated repolarization reserve. LQT2 patients are particularly vulnerable to sudden auditory stimuli during rest, reflecting the relative dependence of IKr on sympathetic tone and the exquisite sensitivity of hERG channels to pharmacological blockade [6, 20].

### 3.3 Mechanistic Complexity: Beyond Simple Loss or Gain of Function

The discovery that certain SCN5A variants confer simultaneous loss‐of‐function and gain‐of‐function electrophysiological effectsgenerating both a Brugada syndrome phenotype and a LQTS phenotype within the same individual—represents one of the more conceptually challenging revelations of recent genetic cardiology. Neubauer et al. demonstrated this dual mechanism in a SCN5A N1774H variant family, in which patch‐clamp experiments on transfected TsA‐201 cells revealed reduced peak sodium current alongside augmented late INa, with heterozygous carriers uniformly demonstrating QTc prolongation, and with the loss‐of‐function component of the variant providing a molecular substrate analogous to Brugada syndrome at the channel level [21]. The findings challenge the prevailing notion that SCN5A mutations are either gain‐of‐function (causing LQT3) or loss‐of‐function (causing Brugada syndrome), and they underscore the importance of comprehensive functional characterization before clinical interpretation of novel variants [16].

At the level of post‐translational regulation, the picture is still more complex. Yoshinaga et al. demonstrated that variants in NAA10—a gene encoding an acetyltransferase involved in N‐terminal acetylation—cause profound repolarization abnormalities via a dual mechanism: augmentation of the late sodium current and simultaneous suppression of IKs [22]. Human induced pluripotent stem cell–derived cardiomyocytes (iPSC‐CMs) from carriers of the NAA10 R4S/Y variant exhibited action potential duration prolongation, irregular automaticity, and impaired contractile force, all of which were partially rescued by gene therapy approaches targeting the variant. The extension of these findings to a patient kindred with severe LQTS and cardiomyopathy provides important translational validation, though it should be acknowledged that iPSC‐CM models—while invaluable—differ from adult cardiomyocytes in maturation state, action potential morphology, and metabolic profile, constraints that must be borne in mind when extrapolating their electrophysiological findings to clinical endpoints [22].

### 3.4 Molecular Forensics: Channelopathies and Sudden Unexplained Nocturnal Death

Perhaps the most sobering application of ion channel genetics is in the postmortem context. Chaloemthanetphong et al. performed whole‐exome sequencing on 98 victims of sudden unexplained nocturnal death syndrome (SUNDS) in Thailand, identifying pathogenic or likely pathogenic SCN5A variants—including the novel Thai‐incident variant A665S and the previously described R965C—in approximately 5% of cases [23]. The electrostatic surface modeling applied to the A665S and R965C variants provided mechanistic plausibility by demonstrating substantial alterations in Nav1.5 gating behavior consistent with a Brugada‐like or LQT3‐like repolarization phenotype; the R179Q variant did not demonstrate substantial electrostatic surface alteration in the same modeling analysis. These data underscore a point that bears repeating: among unexplained sudden deaths in young adults, a proportion that clinical workup will never identify can be attributed post hoc to ion channel dysfunction, reinforcing the argument for cascade genetic screening in first‐degree relatives of all SCD victims [5, 24, 25].

### 3.5 Late Sodium Current as a Therapeutic Target

The identification of the late sodium current as a shared pathological mechanism across multiple genetic and acquired repolarization disorders has had direct therapeutic implications, which are explored more fully in subsequent sections. What deserves emphasis at the molecular level is that the pharmacological suppression of INaL by sodium channel blockers such as mexiletine does not uniformly shorten the QT interval across all LQTS subtypes. Crotti et al. demonstrated, in a carefully designed translational study combining iPSC‐CM electrophysiology, transgenic rabbit models, and a clinical cohort of 96 LQT2 patients, that mexiletine achieved QTc shortening of ≥ 40 ms in 65%–75% of LQT2 patients—a finding initially counterintuitive given that LQT2 is caused by IKr loss of function rather than INaL gain of function [18]. The proposed mechanism involves compensatory action of mexiletine on residual late sodium current that contributes to the already‐prolonged action potential in IKr‐deficient myocytes, combined with indirect effects on calcium homeostasis. The strong inverse correlation between baseline QTc and the magnitude of QTc shortening (r = −0.8) further suggests that the degree of repolarization derangement at baseline partially predicts therapeutic responsiveness—a finding with potential implications for patient selection in clinical practice [18].

## 4 Acquired and Environmental Risk Factors for QT Prolongation

### 4.1 The Pharmacological Burden

Drug‐induced QT prolongation is, in most clinical settings, a substantially more common problem than its congenital counterpart. The hERG channel—simultaneously the molecular target through which IKr mediates repolarization and a promiscuous binder of structurally diverse drugs—is exquisitely vulnerable to pharmacological blockade [2, 26]. The list of implicated agents spans psychiatry, infectious disease, oncology, and gastroenterology: antipsychotics, certain antidepressants, fluoroquinolones, macrolides, antifungals, antiemetics, and prokinetics, among many others [6, 9]. The challenge is not identifying the individual drug‐QT relationships, which are reasonably well‐characterized for most agents; rather, it is predicting which patients, receiving which combinations of drugs, in which clinical context, will cross the threshold into clinically dangerous QTc prolongation.

Albekairy et al. addressed this challenge from an acute care perspective, examining a cross‐sectional cohort of hospitalized patients receiving non‐cardiac drugs and classifying ECGs using a QT nomogram approach [27]. Their finding that proton pump inhibitors as a class accounted for 51% of QTc‐prolongation cases in their sample is striking and somewhat underappreciated in clinical practice, with esomeprazole identified as the single most common agent at 46.5% of all cases, where concern typically focuses on fluoroquinolones and antipsychotics. The antimicrobial agents and antihistamines, by contrast, exhibited higher nomogram risk classifications even when QTc absolute values were less dramatically elevated, suggesting that the pattern of QT prolongation matters as much as its magnitude—a nuance that scalar QTc thresholds fail to capture [27].

### 4.2 Nutritional Substances, Over‐the‐Counter Products, and Gene–Nutrient Interactions

A dimension of pharmacological risk that has received insufficient attention in clinical guidelines—and that operates through mechanisms distinct from those of prescription medications—concerns the electrophysiological consequences of commonly consumed nutritional substances and over‐the‐counter products. Grapefruit juice contains furanocoumarins that irreversibly inhibit intestinal CYP3A4, substantially increasing plasma concentrations of hERG‐blocking drugs—in some cases by two‐ to threefold—thereby converting a sub‐therapeutic QT‐prolonging drug exposure into a clinically dangerous one [28]. Licorice, consumed in candy, herbal preparations, and certain traditional medicine formulations, contains glycyrrhizinic acid, which induces pseudohyperaldosteronism with resultant hypokalemia, an independent QT‐prolonging mechanism that compounds the effects of co‐administered QT‐active drugs. Energy drinks, through their caffeine and taurine content combined with their electrolyte composition, have been associated with QTc prolongation in population‐based and case‐control studies, particularly in younger consumers [29]. The QT‐prolonging potential of herbal and over‐the‐counter supplements—including high‐dose licorice root, St. John's Wort, and several traditional Chinese and Ayurvedic preparations—is documented but inadequately incorporated into standard pre‐prescription QT risk assessments [30, 31, 32].

The gene–nutrient interaction dimension of this problem is perhaps its most clinically under‐recognized aspect. Common pharmacogenomic variants in CYP3A4, CYP3A5, and drug transporter genes including ABCB1 and SLCO1B1 determine the degree of pharmacokinetic interaction between dietary CYP inhibitors and co‐administered drugs, meaning that the same grapefruit juice consumption may have negligible QT consequences in one patient and dangerous drug‐interaction consequences in another with relevant variant alleles [33]. This gene–nutrient–drug triad represents a mechanistically coherent but clinically neglected tier of personalized QT risk assessment. Mazzanti et al. provided a comprehensive mechanistic and clinical synthesis of nutritional contributions to arrhythmic risk in LQTS, documenting that dietary and supplement exposures are not marginal contributors but can be clinically decisive in patients who already carry a genetic predisposition to repolarization abnormality [28]. The practical implication is that dietary and supplement history—including consumption of grapefruit juice, herbal products, licorice‐containing preparations, and high‐dose caffeine formulations—should be routinely assessed in all patients with known or suspected LQTS and in any patient receiving QT‐prolonging pharmacotherapy.

### 4.3 Drug–Disease Interactions and Polypharmacy

The risk carried by any single QT‐prolonging drug does not exist in isolation from the clinical context in which it is prescribed. Wongsalap et al. examined a retrospective cohort of atrial fibrillation patients receiving intravenous amiodarone—an agent whose QT‐prolonging properties are well‐recognized but whose benefit in AF rate control often overrides concern in acute settings—and found that diabetes mellitus, prior stroke, and concurrent administration of antipsychotic or anticholinergic medications were independent predictors of amiodarone‐induced TdP [34]. The interaction between systemic illness and drug‐induced QT prolongation is mechanistically plausible: diabetic cardiomyopathy is associated with intrinsic repolarization reserve impairment; autonomic neuropathy alters the sympathovagal balance that modulates repolarization dynamics; and polypharmacy in complex medical patients creates pharmacokinetic and pharmacodynamic interactions that no single drug monograph can fully anticipate [9].

The drug–gene interaction layer adds yet another dimension of complexity. Ferreira et al. documented that among 195 genetically diagnosed and clinically treated LQTS patients prescribed QT‐prolonging psychiatric medications, only 14 (7%) experienced a breakthrough cardiac event after specialist evaluation, and of these, only 3 occurred while on psychiatric medications—all in the setting of concurrent established arrhythmia triggers including treatment non‐compliance and electrolyte abnormalities—indicating that appropriately managed LQTS patients can receive necessary psychiatric pharmacotherapy without a substantially elevated arrhythmic event rate [35]. This finding is particularly clinically important because patients with LQTS carry both a genetic propensity for repolarization abnormalities and, not infrequently, comorbid anxiety and depression that necessitate psychiatric pharmacotherapy. The interaction is not merely additive; it may be synergistic in the context of genetic repolarization reserve that is already compromised at baseline [2, 35]. Managing this interaction demands interdisciplinary communication between cardiologists, psychiatrists, and clinical pharmacologists—a level of coordination that does not reliably exist in most health systems [6].

### 4.4 Electrolyte Disturbances and the Metabolic Environment

Hypokalemia and hypomagnesemia independently prolong the QTc by reducing the extracellular potassium concentration gradient that normally drives IKr outward current, and by impairing the stabilization of membrane channels, respectively [2, 9]. In hospitalized patients, these disturbances are extraordinarily common—encountered in virtually every ICU cohort studied—and their combination with QT‐prolonging drugs creates a multiplicative, rather than simply additive, risk profile. Platonov et al., analyzing LQT2 mutation carriers with apparently normal QTc values, demonstrated that even in the absence of overt QTc prolongation, specific T‐wave morphological features (flat, notched, or negative patterns) and mutation topology (pore‐region variants) independently predicted arrhythmic events [36]. This finding implies that genetically compromised repolarization reserve can remain electrographically concealed under physiological conditions but may be unmasked by the superimposed metabolic stresses of hospitalization—a paradigm with direct implications for perioperative and critical care management.

### 4.5 Metabolic Syndrome, Insulin Resistance, and Adiposity as Emerging QT Risk Factors

It is important to acknowledge at the outset that the mechanistic plausibility of the following associations is substantial and the epidemiological evidence is consistent, but the evidence linking insulin resistance, NAFLD, and regional adiposity to QT prolongation is derived predominantly from observational cohort studies—capable of establishing association and identifying biological candidates for causal pathways, but not of demonstrating causality in the interventional sense. A body of evidence has been building, largely outside the specialized inherited arrhythmia literature, that the metabolic phenotypes pervasive in modern populations independently prolong ventricular repolarization in individuals with no prior cardiac diagnosis.

The mechanistic plausibility of an insulin resistance–QT prolongation relationship is substantial. Hyperinsulinemia and insulin resistance alter cardiac autonomic tone, reduce cardiac vagal modulation, and promote a systemic inflammatory and oxidative stress milieu that impairs membrane channel function [10, 37]. Insulin resistance is also associated with hypokalemia through its effects on sodium‐potassium ATPase activity, and with dyslipidemia‐driven alterations in sarcolemmal membrane composition that modify ion channel gating kinetics. The quantitative synthesis by Mobasheri‐Shiri et al.—a systematic review and meta‐analysis drawing on multiple population‐based datasets—confirmed a statistically significant positive association between insulin resistance indices and QT interval duration [10]. The magnitude of QT prolongation attributable to insulin resistance in that analysis, while modest at the individual level, has population‐scale implications given the extraordinary prevalence of insulin resistance in contemporary adult populations worldwide.

The hepatic dimension of metabolic QT risk has been characterized with particular rigor in Iranian population‐based data. Naderi et al. reported findings from the Fasa Cohort Study (FACS) demonstrating that nonalcoholic fatty liver disease (NAFLD) was independently associated with QTc prolongation after adjustment for established confounders including age, sex, body mass index, diabetes, and medication use [11]. This finding is mechanistically coherent: NAFLD is deeply intertwined with insulin resistance and systemic inflammatory activation, and it is associated with autonomic dysfunction, elevated plasma free fatty acids, and altered bile acid metabolism—all of which have documented effects on cardiac repolarization. Critically, the association persisted after exclusion of individuals on QT‐prolonging medications, suggesting that NAFLD exerts a pharmacology‐independent effect on QTc [11]. NAFLD affects an estimated 25%–30% of adults globally [38], and if it independently contributes to QT prolongation at even a modest magnitude, it represents a genuinely population‐relevant risk modifier that current QT risk tools have not yet incorporated.

The distribution of body fat, rather than total adiposity alone, further modulates QTc in ways that aggregate BMI metrics do not capture. Yazdanpanah et al. examined the relationship between fat mass in specific anatomical regions and QTc in a large community sample, finding that trunk and upper body fat mass showed stronger associations with QTc prolongation than lower limb fat, consistent with the known differential cardiovascular risk profiles associated with visceral versus peripheral adiposity [12]. The practical implication is that waist circumference or waist‐to‐hip ratio may be more informative QT risk predictors than BMI alone in community screening contexts.

**FIGURE 2:**
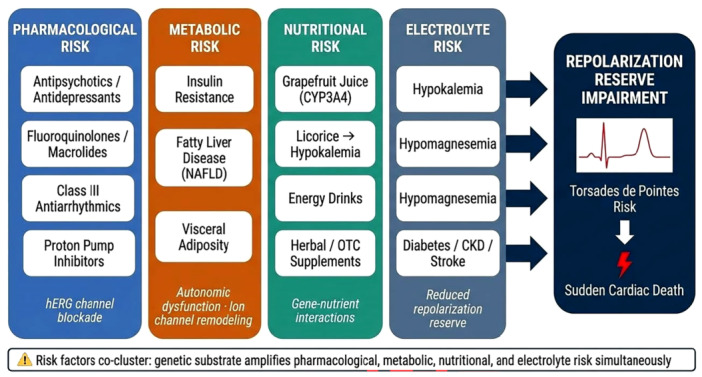
Multi‐factorial risk landscape for QT prolongation and repolarization reserve impairment. Four converging risk domains—pharmacological, metabolic, nutritional/supplement‐related, and systemic/electrolyte—compound repolarization reserve impairment through distinct but interacting mechanisms, collectively predisposing to torsades de pointes and sudden cardiac death. The nutritional tier encompasses grapefruit juice–mediated CYP3A4 inhibition, licorice‐induced pseudohyperaldosteronism with hypokalemia, energy drink constituents, and herbal/over‐the‐counter preparations; gene–nutrient interactions further modulate the magnitude of risk within each domain. Risk factors co‐cluster in the same individuals, and the underlying genetic substrate amplifies the impact of all four acquired risk tiers simultaneously. CYP = cytochrome P450, CKD = chronic kidney disease, NAFLD = nonalcoholic fatty liver disease, OTC = over‐the‐counter.

Taken together, the emerging metabolic risk literature paints a picture of QT prolongation as substantially more than a channelopathy and drug safety problem — it is also a cardiovascular consequence of the global metabolic syndrome epidemic. The substantial overlap between insulin resistance, NAFLD, and visceral adiposity means that these risk factors do not operate in parallel isolation; they cluster in the same individuals, compounding repolarization reserve impairment in ways that no single factor alone fully accounts for. Whether correction of these metabolic risk factors through lifestyle intervention, pharmacological therapy, or weight loss translates into measurable QTc shortening and reduced arrhythmic risk has not been tested in prospective interventional designs, and this represents the critical next step for translating these observational findings into actionable clinical guidance [10, 11, 12]. The convergence of these three risk domains and their joint contribution to repolarization reserve impairment is depicted in Figure 2. Future QT risk models—both clinical instruments and AI prediction platforms—will need to incorporate these metabolic variables explicitly if they aspire to accurate risk estimation in the general population.

The metabolic risk evidence reviewed above complements the nutritional risk data discussed in Section 4.2: in both cases, biologically plausible mechanisms are supported by associational evidence, but the field currently lacks prospective interventional data demonstrating that correcting the exposure reduces QTc or arrhythmic event rates. Both dimensions should therefore be incorporated into QT risk frameworks with appropriate epistemic caution, informing clinical vigilance while the prospective evidence base is developed.

## 5 Risk Stratification in LQTS and Drug‐Induced QT Prolongation: Beyond the QTc

### 5.1 The Foundational Problem: QTc Formula Validity

Before any risk stratification framework that depends on the QTc can be meaningfully evaluated, a question that receives insufficient clinical attention must be addressed: which QT correction formula should be used, and does it matter? The Bazett formula—which divides the raw QT interval by the square root of the R‐R interval—has dominated clinical and research practice for nearly a century by virtue of historical precedent rather than demonstrated superiority. Its well‐documented overcorrection at high heart rates and undercorrection at slow heart rates systematically distorts QTc values at the extremes of heart rate distribution, potentially misclassifying risk in precisely those clinical contexts—exercise, bradycardia, fever, autonomic dysfunction—where accurate repolarization assessment is most consequential [15, 39, 40].

Yazdanpanah et al. addressed this problem directly in the Fasa PERSIAN Cohort Study, evaluating multiple QT correction formulas—including Bazett, Fridericia, Framingham, and Hodges—against resting 12‐lead ECGs in a large non‐hospitalized Iranian community population [15]. Their analysis identified formula‐dependent differences in QTc distributions and in the prevalence of QTc prolongation at standard thresholds—differences that are not merely statistically trivial but potentially clinically consequential. The Fridericia formula performed comparably or superiorly to Bazett in that community cohort [15]. This finding echoes the mechanistic criticisms of Bazett that the pharmacological literature has articulated for years—and it has practical consequences for the AI prediction models discussed in Section 7, most of which use QTc thresholds derived from Bazett‐corrected intervals as their primary outcome definition, potentially introducing systematic measurement error from the outset [15, 39].

### 5.2 The Inadequacy of QTc Alone

The persistent institutional reliance on simple QTc thresholds—most commonly ≥ 500 ms as a clinical action trigger—reflects the practical appeal of a single numerical value rather than any compelling evidence that this threshold reliably identifies patients at imminent risk. As a mounting body of evidence demonstrates, the QTc is necessary but decidedly insufficient as a risk stratification instrument [36, 41, 42]. Genotype‐positive patients with normal‐range QTc values can and do experience life‐threatening arrhythmic events, as Goldenberg et al. established in the landmark International LQTS Registry analysis showing that even within the normal‐QTc stratum, LQT2 female carriers harbored a substantially elevated event risk [43]. Conversely, patients with QTc values well above 500 ms can remain asymptomatic for decades on appropriately dosed beta‐blocker therapy.

The recognition that T‐wave morphology carries independent prognostic information—beyond what QTc conveys—has been building for years but has gained new empirical weight from recent cohort studies [36, 44]. Flat or notched T‐wave configurations, particularly in leads II and V5, reflect heterogeneous repolarization across the myocardial wall, a substrate mechanistically conducive to triggered arrhythmia. The combination of abnormal T‐wave morphology and pore‐region KCNH2 mutations generated a threefold increase in arrhythmic event risk compared to LQT2 carriers without these features, even in the setting of QTc values that clinical algorithms would conventionally classify as borderline [36].

### 5.3 Genotype, Sex, and the Lifecycle of Risk

Risk in LQTS is not static across a patient's lifetime, and its determinants are not uniform across genotypes. Lippert et al., reporting on a large multicenter German pediatric cohort, confirmed that QTc ≥ 500 ms (HR 2.9), LQT3 genotype (HR 2.5), history of syncope (HR 3.0), and absence of cardiac medication (HR 9.5) were the strongest independent predictors of life‐threatening arrhythmic events in children and adolescents [42]. The female predominance of risk in LQT2 has long been recognized but mechanistically remains incompletely explained; sex hormone modulation of IKr expression and autonomic nervous system tone are the most cited candidates [2, 6]. In LQT1, the risk of arrhythmic events is greatest in childhood and adolescence, while LQT3 carries disproportionate event risk in adulthood and during sleep—consistent with the dominant role of increased INaL at slow heart rates [6, 19].

### 5.4 Dynamic Risk Reassessment and the M‐FACT Framework

**FIGURE 3:**
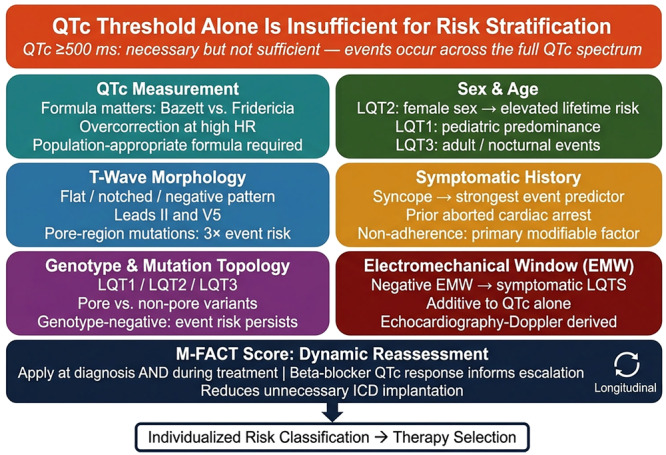
Multi‐parameter risk stratification framework for long QT syndrome. QTc threshold alone is insufficient for clinical risk classification; six independently validated parameters — QTc formula selection, T‐wave morphology, genotype and mutation topology, sex and age, symptomatic history, and the electromechanical window—contribute additive prognostic information. Dynamic application of the M‐FACT score during treatment, rather than a single baseline assessment, reduces inappropriate device escalation without compromising patient safety. EMW = electromechanical window, HR = heart rate, ICD = implantable cardioverter‐defibrillator.

One of the conceptually most important contributions of recent primary research is the demonstration that LQTS risk is not fixed at the time of diagnosis but evolves in response to treatment and intercurrent events. Dusi et al. developed and applied the M‐FACT score—a composite clinical risk instrument incorporating QTc level (scored as one point for 500–550 ms and two points for > 550 ms), age below 20 years, syncope on beta‐blocker therapy, and prior aborted cardiac arrest—to a cohort of 946 LQT1, LQT2, and LQT3 patients followed for a mean of 7 years [41]. Critically, the M‐FACT score was applied both at baseline and dynamically during follow‐up, with therapeutic decisions informed by reassessment of QTc response to beta‐blocker therapy. This dynamic approach reduced ICD implantation rates to 3% without a corresponding increase in life‐threatening arrhythmic events, challenging the prevailing assumption that high‐risk LQTS requires reflexive device therapy irrespective of pharmacological response [41]. The multi‐parameter stratification framework integrating these independent prognostic variables is depicted schematically in Figure 3.

The electromechanical window (EMW)—the interval between the end of mechanical systole (aortic valve closure by Doppler) and the end of electrical systole (end of the T‐wave)—has emerged as an additional stratification parameter with notable discriminatory power. Sugrue et al. demonstrated that a negative EMW was significantly more pronounced in symptomatic compared to asymptomatic LQTS patients, and that EMW provided additive stratification information beyond QTc alone [45].

A complementary and mechanistically important extension of the EMW concept concerns its temporal dynamics as an arrhythmia precursor. Deissler et al. demonstrated that temporary accentuation of EMW negativity—a transient deepening of the negative EMW value over successive cardiac cycles—serves as a marker of impending ventricular tachycardia in patients with congenital LQTS and drug‐induced QT prolongation [46]. In their prospective serial analysis, episodes of transiently amplified EMW negativity preceded documented TdP events by a measurable interval, raising the conceptually important possibility that continuous EMW monitoring—rather than single‐point EMW measurement—could function as an early warning system for imminent arrhythmic events. This temporal dimension of EMW risk is not captured by any static risk score, including the M‐FACT instrument, and it suggests that the future role of this parameter may lie in continuous ambulatory surveillance rather than one‐time clinical assessment. Whether automated detection of EMW variability can be integrated into echocardiographic monitoring platforms or derived from multimodal wearable signal processing remains to be established, but the mechanistic case for its investigation as a dynamic arrhythmia biomarker is now considerably strengthened.

### 5.5 Genotype‐Negative LQTS: A Diagnostic and Stratification Challenge

Karlinski et al. examined a large Mayo Clinic cohort of 2206 LQTS patients evaluated over a 24‐year period and found that genotype‐negative cases constituted less than 2% of the cohort under standard genetic testing panels [47]. Among these, approximately 35% received a molecular diagnosis when advanced next‐generation sequencing was applied. The remaining genotype‐negative patients presented a particularly challenging stratification problem: their clinical event rates were comparable to genotype‐positive patients, yet current tools for genotype‐specific risk scoring are inapplicable in their absence [47]. This creates a genuine gap in the stratification framework, one that AI‐based ECG phenotyping may eventually help to bridge.

### 5.6 Asymptomatic LQTS: Understanding the Benign Majority

A counterpoint to the foregoing narrative of elevated risk comes from the large observational cohort reported by Rudquist et al., in which 74% of LQTS patients were asymptomatic at first clinical evaluation and experienced a breakthrough cardiac event rate of only 1% over an 8‐year follow‐up period [48]. Of the breakthrough cardiac events that did occur, 38% arose in patients who were non‐adherent to prescribed therapy at the time of the event. The data suggest that for the majority of LQTS patients presenting asymptomatically, the natural history under adequate treatment is comparatively benign, and that the principal modifiable determinant of adverse outcomes in this group is not the underlying genotype but rather treatment adherence and avoidance of QT‐prolonging triggers [48].

The concept of asymptomatic LQTS is further refined by the recently characterized phenomenon of nonpenetrant LQTS—a phenotypically distinct subset of genotype‐positive individuals who lack not only symptoms but any objective electrocardiographic or cardiac evidence of abnormal repolarization on any assessment. Rudquist et al. examined the prevalence, spectrum, and outcomes of this nonpenetrant phenotype in a large genotype‐confirmed LQTS cohort, finding that a meaningful proportion of mutation carriers fulfilled criteria for complete nonpenetrance, with QTc values consistently within the normal range across serial assessments and an arrhythmic event rate approaching that of the general population [49]. These findings carry significant clinical implications: they establish that the genetic substrate alone—without any phenotypic electrocardiographic expression—carries a substantially lower arrhythmic risk than genotype‐positive LQTS with any degree of QTc prolongation, and they argue against reflexive pharmacological intervention in genotype‐positive family members who lack any electrocardiographic disease expression. Distinguishing true nonpenetrance from fluctuating low‐level penetrance—which may be unmasked by fever, drug exposure, or electrolyte disturbance—remains an incompletely resolved clinical challenge, underscoring the need for longitudinal electrocardiographic surveillance even in apparently nonpenetrant carriers.

The complementary long‐term outcome data from Tavačová et al., examining a pediatric Czech LQTS cohort followed for up to 20 years, reinforced the prognostic importance of beta‐blocker selection, with non‐selective agents (nadolol) demonstrating superiority over beta‐1 selective blockers in LQT1 and LQT2 [1, 50]. Overall survival reached 97.7% at five years on optimally dosed non‐selective beta‐blocker therapy, with freedom from major arrhythmic events reaching 93.9% at five years—reassuring figures that should temper any reflexive escalation to device therapy in appropriately managed patients [50].

## 6 Clinical Management of LQTS and QT Prolongation: From Empirical Therapy to Precision Medicine

### 6.1 Beta‐Blockers as the Cornerstone

For most LQTS patients, non‐selective beta‐adrenoceptor blockade remains the foundational treatment, and the evidence for its efficacy—particularly in LQT1 and LQT2—is among the most consistently replicated findings in the field [1, 50, 51]. Beta‐blockers attenuate adrenergically mediated increases in heart rate and IKs‐dependent shortening of the QT interval, and they suppress catecholamine‐triggered EADs that may initiate TdP. In LQT1, where arrhythmias are almost exclusively triggered by exercise or emotional stress, nadolol achieves event reduction exceeding 90% in compliant patients [1]. In LQT3, however, the efficacy of beta‐blockade is more circumscribed. Because LQT3 arrhythmias occur predominantly at rest and during sleep, the sympatholytic mechanism of beta‐blockers provides limited protection [19].

### 6.2 Mexiletine: Gene‐Specific Therapy in LQT3 and Beyond

Mazzanti et al. provided the first substantive clinical evidence that mexiletine—a class IB sodium channel blocker with selectivity for late INa at clinically achievable concentrations—prevents life‐threatening arrhythmic events in LQT3 patients [19]. In 34 consecutive LQT3 patients followed for a median of 36 months, mexiletine reduced the annualized arrhythmic event rate from 10.3 to 0.7 events per 100 patient‐years, a 93% relative risk reduction accompanied by a mean QTc shortening of 63 ms. The biological plausibility of this intervention is straightforward: by blocking the gain‐of‐function late INa that is the direct consequence of SCN5A mutations in LQT3, mexiletine addresses the molecular lesion rather than merely compensating for its downstream effects [19, 52].

The subsequent demonstration by Crotti et al. that mexiletine is effective—though by a mechanistically distinct pathway—in a substantial proportion of LQT2 patients substantially expands the therapeutic scope of this old drug [18]. Whether the 25%–35% of LQT2 non‐responders carry distinguishing clinical or genetic features that could guide patient selection remains an open and practically important question [18].

### 6.3 Left Cardiac Sympathetic Denervation

**FIGURE 4:**
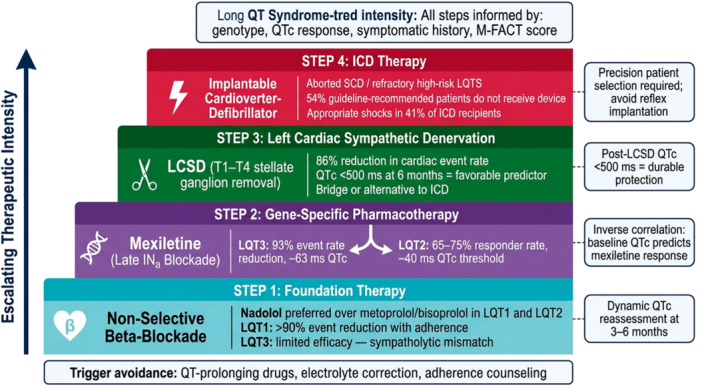
Hierarchical therapeutic escalation algorithm for long QT syndrome. Management proceeds stepwise from non‐selective beta‐blockade through gene‐specific pharmacotherapy with mexiletine, left cardiac sympathetic denervation, and implantable cardioverter‐defibrillator implantation, with each escalation governed by dynamic reassessment of QTc response, symptomatic status, and composite risk score. Genotype‐specific efficacy profiles and response predictors are indicated at each step. ICD = implantable cardioverter‐defibrillator, LCSD = left cardiac sympathetic denervation, INa = sodium current, SCD = sudden cardiac death.

For patients in whom pharmacological therapy is insufficient or not tolerated, left cardiac sympathetic denervation (LCSD) represents a decades‐tested non‐pharmacological intervention [53]. The historical data from Dusi et al., representing 50 years of experience with LCSD at a single center, are among the most compelling non‐randomized intervention data available for any therapy in inherited arrhythmia. A mean 86% reduction in annualized cardiac event rate following LCSD, accompanied by significant QTc shortening in patients with baseline QTc ≥ 500 ms, and a reliable predictor of favorable long‐term outcome—namely, post‐LCSD QTc < 500 ms at six months — provides a stratification framework for patient selection that is practically actionable [53]. The hierarchical escalation framework integrating genotype‐specific therapy selection with dynamic risk reassessment is presented in Figure 4.

### 6.4 ICD Therapy: Appropriate Use and the Problem of Overimplantation

The ICD occupies a paradoxical position in LQTS management: it is the most reliably effective treatment for preventing arrhythmic death, yet it carries its own burden of inappropriate shocks, lead complications, psychological morbidity, and device‐related infections that must be weighed against the probabilistic benefit it confers [54, 55]. Neves et al. examined consequences of non‐adherence to guideline‐based ICD recommendations in a merged cohort of 2861 LQTS patients, finding that among patients who met ESC 2022 class I or IIa ICD indications, 54% did not receive a device [54]. In the ICD‐deferred group, only 4% experienced non‐lethal breakthrough cardiac events, while 41% of those who received an ICD experienced at least one appropriate ventricular fibrillation‐terminating shock—findings that support a precision medicine approach over reflex guideline‐driven implantation [54].

### 6.5 Pharmacological Complexity in Real‐World Clinical Care

Managing LQTS in the real world requires navigating multiple competing considerations. Many patients carry comorbidities requiring QT‐prolonging medications, as the data from Ferreira et al. illustrate in the context of psychiatric pharmacotherapy [35]. Others present late in life, when the accumulated burden of metabolic comorbidities has modified the baseline electrophysiological substrate in ways that no current clinical risk tool explicitly accounts for [10, 11]. The observation from Rohatgi et al. that 92% of patients across the entire spectrum remained event‐free over a median follow‐up of 6.7 years—while three LQT3 patients ultimately required cardiac transplantation—maps the full range from reassuringly favorable to catastrophically refractory outcomes that any comprehensive management framework must accommodate [51].

## 7 Artificial Intelligence‐Based Risk Prediction in QT Prolongation and Sudden Cardiac Death

### 7.1 The Limitations of Conventional Risk Scores

The Tisdale score and the RISQ‐PATH instrument were for many years the primary decision‐support tools available to clinicians attempting to anticipate dangerous QTc changes in complex inpatients [56]. Both instruments incorporate readily available clinical variables and achieve reasonable discrimination in the populations in which they were developed [56, 57]. Their limitations are substantial: poor performance when applied to heterogeneous populations outside their derivation cohorts; static rather than dynamic risk capture; and inability to extract subtle morphological information from the ECG signal [2, 58]. Conventional risk scores also lack metabolic variables, meaning their discriminatory performance may be systematically degraded in populations where metabolic syndrome is prevalent—precisely those constituting the majority of modern hospital admissions [11, 12, 15].

### 7.2 Machine Learning Models for Drug‐Induced QT Prolongation Prediction

Jing et al. compared an XGBoost machine learning model against the Tisdale and RISQ‐PATH clinical risk scores in a retrospective cohort drawn from the Geisinger Health System, comprising more than 300,000 ECG records from patients initiating QT‐prolonging medications [58]. The XGBoost model achieved an AUROC of 0.859, substantially exceeding the RISQ‐PATH AUROC of 0.701 in the overall population and the Tisdale score AUROC of 0.770 in the inpatient‐predominant subgroup. These performance advantages were demonstrated in a retrospective cohort design; prospective clinical effectiveness studies demonstrating that algorithmic risk prediction actually changes prescribing decisions and improves patient outcomes have not yet been reported for this or comparable platforms. The addition of ECG signal features to electronic health record variables provided incremental discriminatory improvement beyond either data source alone [58, 59, 60].

A measurement consideration that applies to this and related studies deserves explicit acknowledgment. The QTc ≥ 500 ms outcome threshold used in the Jing et al. model was defined using Bazett‐corrected QT intervals. As Yazdanpanah et al. demonstrated, however, the choice of correction formula systematically affects QTc distributions and threshold prevalence [15]. Models trained on Bazett‐derived QTc thresholds may harbor formula‐dependent classification error—a source of systematic bias that has received little attention in the AI‐ECG literature but has direct implications for model calibration and clinical deployment accuracy.

Zhang et al. applied a convolutional neural network (CNN)—termed QTNet—to the 12‐lead ECG signals of 44 386 outpatients prescribed QT‐prolonging medications, achieving a mean AUROC of 0.802 for predicting drug‐induced LQTS [8]. The survival analysis framework employed, examining risk accumulation over days to weeks following drug initiation, is a methodological advance over studies relying on single point‐in‐time predictions [8, 61]. Whether the ECG features identified by QTNet correspond to interpretable electrocardiographic patterns remains an open question for model transparency [13, 14].

### 7.3 Continuous Outpatient QT Monitoring via Deep Learning

Ansari et al. reported a prospective validation study of a spatial deep learning algorithm—termed 3DRECON‐QT—applied to single‐lead ECG signals from insertable cardiac monitors to reconstruct a spatially aware QT measurement and continuously track high‐risk prolongation events in patients receiving class III antiarrhythmics as outpatients [62, 63, 64]. The clinical implication, if the technology is validated in larger prospective trials, is potentially transformative: the ability to detect dangerous QTc excursions in real time outside hospital settings would substantially reduce the logistical burden of antiarrhythmic initiation [62]. This application fits within a broader trajectory of AI‐powered continuous cardiac monitoring [65, 66, 67].

### 7.4 Deep Learning for Sudden Cardiac Death Risk Stratification

Holmstrom et al. developed and externally validated a 12‐lead ECG–based deep learning model for SCD risk assessment, using a dataset of 1796 out‐of‐hospital SCD cases with prospective external validation in a Ventura County cohort of 714 additional cases [68]. The DL model significantly outperformed a validated six‐variable conventional ECG risk score, and the incremental addition of clinical variables further improved performance in both internal and external cohorts [68]. It bears emphasis that the DL model was derived from community‐based out‐of‐hospital cardiac arrest registries, which capture a population skewed toward older individuals with structural heart disease rather than the younger, genetically defined LQTS patients who dominate most inherited arrhythmia registries; whether performance generalizes to the LQTS‐specific population—where arrhythmic mechanisms differ fundamentally—remains empirically unresolved.

Prifti et al. addressed the LQTS‐specific gap more directly, training a CNN on ECGs from congenital LQTS patients and sotalol‐treated individuals, demonstrating that the model could distinguish ECGs at high arrhythmic risk from drug exposure or LQT2 genotype and could identify ECGs recorded proximate to TdP episodes in serial monitoring [69]. The LQT subtype‐specific signal detected by that model—consistent with the distinct T‐wave morphologies characteristic of LQT1 (broad‐based), LQT2 (low‐amplitude notched), and LQT3 (late‐onset peaked)—suggests that genotype‐aware ECG feature extraction may substantially improve DL model performance in the LQTS population specifically [6, 69].

A particularly consequential AI‐ECG capability demonstrated in recent work is the ability of deep neural network analysis to distinguish patients with congenital LQTS from those with acquired QT prolongation—a clinically critical distinction that has historically been challenging at the bedside. Bos et al. applied a DNN to 12‐lead ECGs from patients with genotype‐confirmed congenital LQTS, healthy controls, and patients with acquired QT prolongation from pharmacological causes, demonstrating that the network reliably classified each group with discriminatory performance substantially exceeding conventional QTc‐based criteria [70]. The DNN appears to exploit the subtle morphological signatures distinguishing genotype‐specific T‐wave patterns of LQT1 (broad‐based), LQT2 (low‐amplitude notched), and LQT3 (late‐peaked) from the more morphologically indiscriminate QT lengthening of hERG‐blocking drug effects. This capability addresses one of the most practically challenging diagnostic scenarios in inherited arrhythmia practice: the patient presenting with markedly prolonged QTc in whom the relative contributions of genetic predisposition and pharmacological exposure are uncertain, and in whom management decisions—including whether to pursue comprehensive genetic testing, restrict the QT‐prolonging drug, and initiate LQTS‐specific therapy—hinge on distinguishing the two mechanisms. As with other DNN‐based ECG tools, prospective clinical validation in diverse populations is required before this diagnostic capability can be reliably deployed as a primary clinical decision‐support instrument.

### 7.5 Multimodal AI: Integrating Diverse Data Sources

**FIGURE 5:**
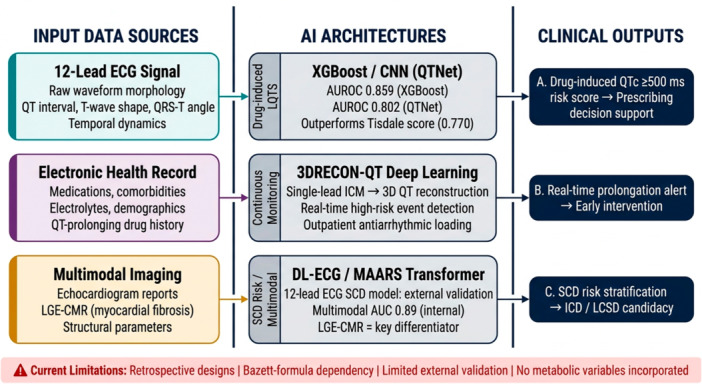
Artificial intelligence‐based risk prediction architectures for QT prolongation and sudden cardiac death. Three input data streams (12‐lead ECG signal, electronic health records, and multimodal imaging) feed four AI architectures operating in parallel: XGBoost/CNN platforms for drug‐induced QT prediction; continuous deep learning monitoring via insertable cardiac monitors; a multimodal transformer network for sudden cardiac death risk stratification; and a deep neural network that distinguishes congenital from acquired QT prolongation at the ECG level. Each architecture produces a distinct clinical output that informs a specific management decision. All four platforms substantially outperform validated clinical risk scores in retrospective cohort benchmarking. Current limitations applicable to all platforms are summarized in the bottom panel. AUROC = area under the receiver operating characteristic curve, DNN = deep neural network, ICM = insertable cardiac monitor, LCSD = left cardiac sympathetic denervation, LGE‐CMR = late gadolinium enhancement cardiac magnetic resonance imaging, SCD = sudden cardiac death.

Lai et al. applied the MAARS transformer‐based neural network to hypertrophic cardiomyopathy patients, integrating electronic health records, echocardiographic reports, and late gadolinium enhancement cardiac MRI, achieving an internal AUC of 0.89 and an external AUC of 0.81 [71]. While HCM is a distinct entity from LQTS [72, 73], the MAARS study is relevant for two reasons: it demonstrates that AI integration of structural, functional, and clinical data substantially outperforms guideline‐based calculators; and it shows such models can be applied fairly across demographic subgroups [71, 74]. The integration of imaging‐derived tissue characteristics proved to be a key differentiating feature, underscoring that predictors of arrhythmic sudden death are not confined to any single data modality [75]. None of the metabolic variables reviewed in Section 4.5 have yet been incorporated into validated AI risk prediction platforms for QT‐related arrhythmia, representing a tractable and scientifically well‐motivated direction for future model development [76]. The architecture, data inputs, and current limitations of the principal AI platforms reviewed here are summarized in Figure 5.

### 7.6 Critical Appraisal of the AI Literature

The enthusiasm surrounding AI‐based arrhythmic risk prediction must be calibrated against several methodological concerns that traverse the studies reviewed here [77, 78]. Retrospective cohort designs predominate, introducing selection bias and retrospective outcome ascertainment susceptible to incomplete capture of soft endpoints [8, 58, 68]. Critically, no published study has yet demonstrated that AI‐based QT risk prediction changes clinical decisions in prospective practice and produces measurable improvements in patient outcomes—as distinguished from demonstrating superior discrimination of outcomes that were observed retrospectively [79]. External validation, where present, often involves cohorts sharing geographic, institutional, or demographic characteristics with the derivation cohort, limiting true generalizability claims [13, 74]. The interpretability of deep learning models remains a fundamental challenge; without understanding which ECG features drive predictions, clinicians cannot assess whether the model exploits biologically meaningful signal or confounding variables [59, 80]. The QTc formula dependency identified by Yazdanpanah et al. [15] adds a further layer: if the QTc threshold outcomes used to label training datasets are formula‐dependent, then apparent ML performance advantages over conventional scores may partly reflect internal consistency against the same formula‐specific outcome definitions rather than formula‐independent clinical validity.

These limitations do not negate the considerable scientific promise of AI‐based QT risk prediction. They do, however, establish that claims of clinical superiority over conventional instruments — while supported in retrospective benchmarking analyses—must be understood as preliminary until confirmed by prospective effectiveness studies. The field's transition from demonstration of discriminatory performance to demonstration of clinical impact represents the central methodological frontier for AI‐ECG research in this domain.

## 8 Integrative Synthesis: Toward a Unified Framework for QT‐Related Arrhythmic Risk

### 8.1 The Case for Multi‐Domain Integration

**FIGURE 6:**
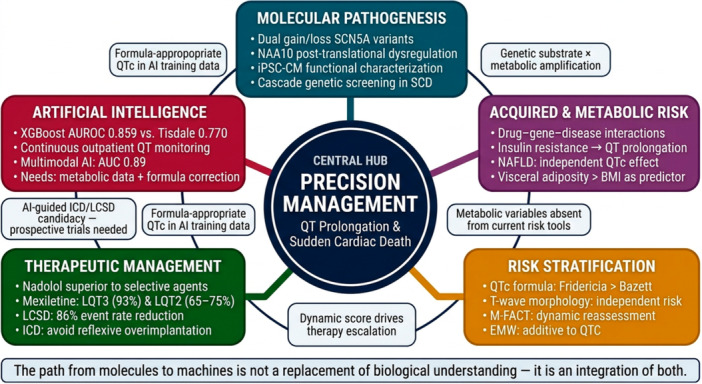
Final integrative summary: A five‐domain framework for precision management of QT prolongation and sudden cardiac death. Five interdependent domains—molecular pathogenesis, acquired and metabolic risk, risk stratification, therapeutic management, and artificial intelligence—converge on precision clinical care. Cross‐domain interactions identified in this review are indicated between adjacent panels. Effective implementation requires bidirectional translation: biological insight must inform algorithmic model design, and computational prediction must be interpreted within its molecular and metabolic context. AUROC = area under the receiver operating characteristic curve, BMI = body mass index, EMW = electromechanical window, ICD = implantable cardioverter‐defibrillator, LCSD = left cardiac sympathetic denervation, NAFLD = nonalcoholic fatty liver disease.

The five research domains reviewed here are not merely conceptually adjacent—they are functionally interdependent in ways that have direct management implications. Molecular genetic testing defines the biological substrate of risk, but its clinical value is substantially determined by the quality of phenotypic classification, the thoroughness of cascade family screening, and the availability of genotype‐specific therapeutic options [2, 6, 47]. Those therapeutic options—mexiletine in LQT3 and LQT2; nadolol over beta‐1 selective agents in LQT1 and LQT2; LCSD as bridge or alternative to ICD—can only be rationally selected if the genetic context is known [18, 19, 50, 53]. Risk stratification must be dynamic and must rest on accurate QTc measurement: a patient who was at high risk at diagnosis and remains at high risk after 6 months of optimal pharmacological therapy warrants different management than one whose QTc has normalized and who has remained asymptomatic [15, 41]. AI‐based models, applied to continuous ambulatory ECG data in the former patient, might detect temporal patterns of QT variability that presage arrhythmic events days before they occur [62, 69]. The functional interdependencies between these five domains are consolidated in Figure 6.

The newly established metabolic and nutritional dimensions of QT risk introduce further levels of integration that have no clear precedent in existing clinical pathways [81]. A patient with NAFLD, insulin resistance, visceral adiposity, and a history of regular energy drink consumption who is prescribed a QT‐prolonging psychiatric medication presents a risk profile that no current clinical scoring system adequately captures. The convergence of pharmacological, genetic, metabolic, and nutritional risk factors in any given patient is the rule in real‐world clinical populations, not the exception; a framework that models only one of these domains at a time will systematically underestimate true cumulative risk. The key evidence synthesized across all five domains is consolidated in Table 1, which provides a structured cross‐reference of study design, population, primary endpoint, and principal finding for each source reviewed. This table is intended to serve as a navigational reference throughout the article.

**TABLE 1:** Multidimensional insights into QT prolongation and long QT syndrome.

ASPECT 1: Molecular and Genetic Mechanisms of QT Prolongation						
Study Design	Population/Model	Gene/Channel Target	Primary Molecular Endpoint	Key Finding	Level of Evidence & Key Limitations	Citation
iPSC‐CM + transgenic model	NAA10 R4S/Y iPSC‐CMs + patient kindred	NAA10 → late INa ↑ /IKs↓	Dual channel dysregulation; contractile force reduction	NAA10 variants cause severe LQTS/CMP; gene therapy rescued phenotype	LoE: Experimental translational study (iPSC + animal + patient kindred). Limitations: iPSC‐CM immaturity; small kindred; single‐center.	[22]
Forensic WES	98 SUNDS post‐mortem cases, Thailand	SCN5A A665S, R179Q, R965C	Electrostatic surface impact of Nav1.5 variants	SCN5A variants in ~5% SUNDS; A665S novel	LoE: Retrospective forensic cohort. Limitations: Single‐country sample; incomplete family history; electrostatic modeling only.	[23]
Retrospective cohort + patch‐clamp	SCN5A N1774H carrier family; TsA‐201 cells	SCN5A N1774H dual LOF/GOF	Patch‐clamp: peak INa ↓ + late INa↑	Dual mechanism: LQTS + Brugada in same family	LoE: Family‐based case series + functional assay. Limitations: Single family; in vitro functional data only.	[21]
Retrospective cohort	34 LQT3 patients	SCN5A GOF (LQT3)	Arrhythmic event rate; QTc shortening	Mexiletine: 93% event rate reduction; QTc −63 ms	LoE: Retrospective before–after cohort. Limitations: No control group; single center; small sample.	[19]
Translational (iPSC + rabbit + clinical)	96 LQT2 patients; iPSC‐CMs; transgenic rabbits	KCNH2 LOF; mexiletine on residual INa/calcium	QTc shortening ≥ 40 ms; iPSC‐CM field potential; APD90	65–75% LQT2 responders; inverse correlation baseline QTc versus ΔQTc (r = −0.8)	LoE: Multi‐pronged translational study (high quality). Limitations: Observational clinical component; no placebo control; single responder threshold definition.	[18]

ASPECT 2: Acquired and Environmental Risk Factors for QT Prolongation						
Study Design	Population/Model	Gene/Channel Target	Primary Molecular Endpoint	Key Finding	Level of Evidence & Key Limitations	Citation
Retrospective cohort; fivefold CV	> 300,000 ECG records, Geisinger Health System	Drug‐induced QTc ≥ 500 ms	AUROC versus Tisdale/RISQ‐PATH	XGBoost AUROC 0.859 vs. RISQ‐PATH 0.701 (overall population) and Tisdale 0.770 (inpatient subgroup)	LoE: Retrospective ML validation. Limitations: Single health system; Bazett formula dependency; no prospective effectiveness data.	[58]
Retrospective cohort	AF patients receiving IV amiodarone, Thailand	TdP with amiodarone; DM, stroke, antipsychotics	TdP incidence; multivariable regression	DM, stroke, concurrent antipsychotics: independent TdP predictors	LoE: Single‐center retrospective cohort. Limitations: Single‐country; small TdP event number; unmeasured confounders.	[34]
Cross‐sectional	Non‐cardiac drug recipients; ICU/wards	PPIs, antimicrobials, antihistamines	QTc ≥ 500 ms; nomogram risk classification	PPIs (esomeprazole) = 51.7% of QTc prolongation cases	LoE: Cross‐sectional (lower LoE). Limitations: Single center; no longitudinal data; possible selection bias.	[27]
Retrospective cohort	LQTS patients receiving psychiatric medications; Mayo Clinic	Psychiatric drug–gene interaction in treated LQTS	Breakthrough cardiac events (BCEs)	Psychiatric medications significantly increased BCE risk in treated LQTS	LoE: Retrospective registry‐based cohort. Limitations: Incomplete medication adherence data; heterogeneous psychiatric diagnoses.	[35]
Retrospective cohort (International LQTS Registry)	154 LQT2 mutation carriers with normal QTc; 1,007 genotype‐negative family member controls	T‐wave morphology; KCNH2 pore mutations; normal QTc	Cardiac events in normal‐QTc LQT2 carriers	Abnormal T‐wave + pore mutations: 3× event risk even with normal QTc	LoE: Large international registry (moderate‐high LoE). Limitations: Retrospective; morphology scoring variability across centers.	[36]

ASPECT 3: Risk Stratification of QT Prolongation						
Study Design	Population	Stratification Tool	Outcome(s)	Key Finding	Level of Evidence & Key Limitations	Ciatation
Retrospective (Mayo Clinic, 24 yr)	2206 LQTS patients	Comprehensive NGS; phenotype‐positive, genotype‐negative definition	Genotype‐negative prevalence; cardiac event rate	Genotype‐negative < 2%; NGS identified cause in 35% on retesting	LoE: Large single‐center registry. Limitations: Single‐institution; long time span with evolving genetic technology.	[47]
Retrospective cohort	LQTS patients diagnosed in childhood; Prague	Schwartz score; QTc; beta‐blocker type (nadolol vs. metoprolol)	MAEs over 5–20 years	Non‐selective BBs superior to selective agents; overall survival 97.7% at 5yr; freedom from MAEs 93.9% at 5yr	LoE: Single‐center retrospective cohort. Limitations: Non‐randomized beta‐blocker assignment; no blinding.	[50]
Retrospective cohort (Mayo Clinic)	1547 LQTS patients, asymptomatic at first evaluation	QTc; sex; genotype; age; symptomatic risk factors	Breakthrough cardiac events (BCEs)	74% asymptomatic; 1% BCE over 8 year; 38% BCEs in non‐adherent patients	LoE: Retrospective registry. Limitations: Single center; adherence data partly self‐reported.	[48]
Prospective cohort	German pediatric LQTS cohort; multicentre	QTc; genotype; sex; age; syncope history	Life‐threatening arrhythmic events	QTc ≥ 500 ms (HR 2.9), LQT3 (HR 2.5), syncope (HR 3.0), absence of cardiac medication (HR 9.5): independent multivariate predictors	LoE: Prospective multicenter (higher LoE). Limitations: Pediatric population only; referral bias.	[42]
Retrospective cohort with dynamic scoring	946 LQT1/2/3 patients; Milan; mean FU 7 year	M‐FACT score (static + dynamic); beta‐blocker QTc response	CEs; aborted cardiac arrest; ICD implantation rate	Dynamic M‐FACT reduced ICD implants to 3% without increasing life‐threatening events	LoE: Single‐center retrospective cohort with prospective‐like scoring. Limitations: Non‐randomized; single‐center referral population.	[41]
Retrospective cohort (International LQTS Registry)	469 genotype‐confirmed LQTS, normal‐range QTc (≤ 440 ms)	QTc within normal range; genotype; sex; syncope	Arrhythmic events including SCD	LQT2 + female sex = highest risk among normal‐QTc genotype‐positive subgroup	LoE: Large international registry. Limitations: Retrospective; ascertainment bias toward symptomatic families.	[43]
Retrospective cohort (Mayo Clinic)	651 LQTS patients; 50 controls; 158 symptomatic	Electromechanical window (EMW) by echo‐Doppler; QTc	Symptomatic versus asymptomatic LQTS status	Negative EMW more profound in symptomatic LQTS; additive to QTc	LoE: Retrospective single‐center case‐control design. Limitations: Echo‐Doppler acquisition variability; needs multicenter validation.	[45]

ASPECT 4: Clinical Management of QT Prolongation/LQTS						
Study Design	Population	Intervention	Primary Outcome	Key Finding	Level of Evidence & Key Limitations	Citation
Retrospective cohort (2 centers)	2861 LQT1/2/3; Mayo + IRCCS Auxologico	ICD versus no ICD; ESC 2022 guideline indications	ICD shocks; BCEs in deferred group	54% guideline‐indicated patients deferred; 4% BCEs in deferred versus 41% ICD shocks	LoE: Multicenter retrospective cohort. Limitations: Non‐randomized; guideline non‐adherence may reflect informed clinical judgment.	[54]
Retrospective cohort (Milan)	946 LQT1/2/3; M‐FACT ≥ 2 subgroup	Dynamic reassessment → LCSD/mexiletine/ICD escalation	CEs during therapy escalation	Structured escalation: CEs in only 14% of high‐risk patients	LoE: Single‐center retrospective cohort. Limitations: Non‐randomized escalation; center‐of‐excellence referral bias.	[41]
Retrospective 50 yr experience	125 LQTS patients undergoing LCSD; mean FU 12.9 year	LCSD (T1–T4 sympathetic chain removal)	Cardiac event rate before/after LCSD; QTc change	86% reduction in annual CE rate; post‐LCSD QTc < 500 ms = excellent outcome predictor	LoE: Retrospective long‐term observational study (single center of excellence). Limitations: No control group; patient selection bias; non‐randomized.	[53]
Translational (iPSC + rabbit + clinical)	96 LQT2 patients; iPSC‐CM; transgenic LQT2 rabbit	Mexiletine in LQT2 (add‐on to beta‐blockers)	QTc shortening ≥ 40 ms; recurrence of arrhythmic events	65–75% responder rate; inverse correlation baseline QTc versus ΔQTc	LoE: Multi‐pronged translational study. Limitations: No placebo control in clinical arm; heterogeneous LQT2 variant types.	[18]
Retrospective cohort (34 patients)	34 consecutive LQT3 patients; Milan	Mexiletine (8 mg/kg/day; median 36 months)	Arrhythmic events before versus after mexiletine; QTc change	93% event rate reduction (10.3 → 0.7/100 PY); QTc −63 ms	LoE: Before–after retrospective cohort (no control group). Limitations: Small sample; single center; absence of placebo comparator.	[19]
Retrospective cohort (Mayo Clinic)	606 LQTS; median FU 6.7yr	Beta‐blockers alone/ICD/LCSD/combination/no therapy	BCEs; death; cardiac transplantation	92% event‐free; three LQT3 patients required transplantation	LoE: Single‐center retrospective cohort. Limitations: Heterogeneous treatment assignment; single‐center referral population.	[51]

ASPECT 5: Artificial Intelligence–Based Risk Prediction						
Study Design	Population/Dataset	AI Architecture	Comparator	Key Performance Metric	Level of Evidence & Key Limitations	Citation
Retrospective; fivefold CV + ext. validation	Geisinger Health System; ~300 000 records	XGBoost; DNN; combined EHR + ECG	Tisdale score; RISQ‐PATH	AUROC 0.859 (XGB); PPV 61.5% vs. 28.3% (RISQ‐PATH) and PPV 54.0% vs. 28.3% (Tisdale) at matched operating points	LoE: Retrospective ML validation. Limitations: Single health system; Bazett formula dependency; no prospective effectiveness trial.	[58]
Retrospective cohort; held‐out + ext. validation	44 386 outpatients, NYU Langone Health	QTNet: CNN on 12‐lead ECG	ECG‐only; QTc‐only; risk‐factor‐only models	Mean AUROC 0.802; survival analysis superiority at day 2+	LoE: Retrospective; external validation present but geographically similar. Limitations: Bazett‐derived outcomes; single US health system.	[8]
Prospective validation; device‐based monitoring	Class III antiarrhythmic initiators; insertable cardiac monitors	3DRECON‐QT: spatial deep learning; single‐lead reconstruction	Standard clinic QTc measurement	Continuous detection of high‐risk QT prolongation events missed by intermittent ECG	LoE: Prospective validation study (higher LoE). Limitations: Real‐world class III antiarrhythmic outpatient cohort *n* = 1,676; requires ICM hardware; no clinical outcome data yet.	[62]
Retrospective + prospective ext. validation	1796 SCD cases (Portland); 714 external (Ventura County)	12‐lead ECG‐based DL model (1827 ECGs)	6‐variable conventional ECG risk score	DL model superior in internal and external cohorts	LoE: Retrospective with prospective external validation (moderate‐high LoE). Limitations: SCD population skewed to older structural heart disease; community SCD not LQTS‐specific.	[68]
Internal + external validation	HCM patients; multicentre	MAARS: transformer‐based multimodal (EHR + echo + LGE‐CMR)	AHA/ACC HCM SCD risk calculator	AUC 0.89 (internal); 0.81 (external); fair across demographic subgroups	LoE: Multicenter internal + external validation. Limitations: HCM‐specific; not validated in LQTS or acquired QT prolongation; LGE‐CMR required.	[71]
Retrospective + prospective validation	ECG pairs: controls; sotalol‐treated; congenital LQTS; French cohort	CNN on 12‐lead ECG; LQT subtype distinction	Standard QTc measurement; manual annotation	CNN distinguishes drug‐induced arrhythmia risk and LQTS subtypes; predicts proximity to TdP in serial monitoring	LoE: Retrospective cohort. Limitations: Single country; moderate cohort size; DNN interpretability not established.	[69]

### 8.2 The Inadequacy of Siloed Clinical Practice

The literature reviewed here exposes the degree to which siloed clinical practice—in which cardiologists make LQTS management decisions without pharmacogenomic guidance, intensivists prescribe QT‐prolonging medications without systematic risk scoring, endocrinologists and hepatologists manage metabolic disease without awareness of its cardiac repolarization consequences, and AI models are developed by data scientists with limited electrophysiological input—produces outcomes that none of the individual disciplines alone would sanction. The data from Ferreira et al. on drug‐gene interactions [35]; the data from Albekairy et al. on unexpected QTc prolongation from under‐recognized drugs [27]; the nutritional interaction evidence synthesized by Mazzanti et al. [28]; the data from Naderi et al. on NAFLD as an independent QT risk factor [11]; and the data from Neves et al. on guideline non‐adherence in ICD implantation [54]—all point in the same direction: the current care model is poorly adapted to the biological complexity of QT‐related arrhythmia risk.

What a unified framework would look like in practice is beginning to come into view. At the molecular level, comprehensive genetic testing should be standard for all suspected congenital LQTS cases and for unexplained SCD victims undergoing molecular autopsy [5, 6, 47]. At the phenotypic level, risk scoring should incorporate QTc (derived from an appropriate correction formula validated in the relevant population [15]. genotype, T‐wave morphology, sex, age, symptomatic history, and EMW, applying these variables dynamically throughout treatment [41, 42, 45]. At the metabolic and nutritional level, clinical assessments should include evaluation of insulin resistance, hepatic steatosis, regional adiposity, and dietary/supplement exposure as independent QT risk modifiers [10, 11, 12, 28]. At the pharmacological level, prescribing algorithms should incorporate ML‐enhanced predictions integrating ECG signal, comorbidity patterns, and genetic data [8, 58]. At the clinical‐technological interface, AI‐powered continuous monitoring platforms should be incorporated into outpatient surveillance of high‐risk individuals [62].

### 8.3 Persistent Gaps and Priority Research Questions

Several empirical gaps are sufficiently important to warrant explicit identification. First, the metabolic and nutritional QT risk literature is observationally coherent but interventionally untested: whether correction of insulin resistance, regression of NAFLD, reduction of visceral adiposity, or elimination of grapefruit and licorice exposure from at‐risk patients translates into measurable QTc shortening and reduced arrhythmic event rates has not been examined in prospective interventional designs. This absence is not a minor gap; it is the critical pathway through which these observational associations would need to travel to justify formal guideline incorporation. Second, the temporal EMW variability data of Deissler et al. [46] must be validated in multicenter prospective cohorts before this dynamic biomarker can be incorporated into routine clinical risk algorithms. Third, AI models for QT‐based SCD risk have demonstrated impressive discriminatory performance in retrospective datasets, but prospective clinical effectiveness studies demonstrating that algorithmic risk prediction actually changes clinical decisions and improves patient outcomes have not been reported for any currently available platform [13, 14, 74]. Fourth, the nonpenetrant LQTS phenotype identified by Rudquist et al. [49] requires further prospective characterization to establish the conditions under which apparent nonpenetrance transitions to phenotypic expression, and the appropriate monitoring interval and intensity for this population. Fifth, the ability of DNN analysis to distinguish congenital from acquired QT prolongation [70] requires validation in prospectively enrolled, ethnically diverse cohorts where this diagnostic uncertainty is clinically common.

The pharmacogenomic dimension of drug‐induced LQTS also deserves more systematic attention. Whether embedding pharmacogenomic data—including variants in CYP3A4, CYP3A5, ABCB1, and KCNH2 itself—into ML‐based QT prediction models would substantially improve their performance remains empirically open [58]. The question of formula‐appropriate QTc thresholds in diverse population groups, raised by the Yazdanpanah et al. data [15], similarly deserves prospective evaluation in the model development and external validation frameworks used by the AI‐ECG field.

### 8.4 Practical Recommendations: Interim Clinical Guidance

Pending the prospective validation and clinical implementation of the integrated framework described above, several evidence‐informed practical recommendations can be offered based on the findings synthesized in this review.

Regarding QTc correction for research and AI model training, the Fridericia formula (QTcF = QT/RR^1^/^3^) should be adopted as the preferred QT correction approach in population‐based research and in the development and training of AI risk prediction models. The population‐based evidence from Yazdanpanah et al. demonstrates that the Bazett formula overcorrects at elevated heart rates and undercorrects at slow heart rates, introducing systematic measurement bias that distorts QTc distributions and threshold‐based outcome definitions [15]. AI models trained on Bazett‐derived QTc thresholds may harbor formula‐dependent classification error that compromises calibration across diverse populations. Until formula‐appropriate QTc standards are universally adopted, AI‐ECG studies should report performance metrics stratified by heart rate quintile and should explicitly state which correction formula was used to define outcome labels in training datasets.

Regarding interim metabolic screening in at‐risk clinical practice, clinicians managing patients who require QT‐prolonging pharmacotherapy—particularly antipsychotics, antidepressants, fluoroquinolones, class III antiarrhythmics, and methadone—should include a structured assessment of metabolic risk as part of pre‐prescription QT risk evaluation. Recommended components include: fasting glucose or HbA1c as a proxy for insulin resistance; liver function tests and hepatic imaging where clinically accessible as a screen for NAFLD; and waist circumference or waist‐to‐hip ratio as an estimate of visceral adiposity. Patients who are positive for two or more of these metabolic risk factors should be considered at elevated baseline QT risk even in the absence of other recognized clinical risk factors, and a lower threshold for baseline and follow‐up ECG monitoring should be applied. These recommendations are not yet supported by prospective interventional trial data but are mechanistically coherent with the observational evidence reviewed here and represent a conservative, low‐cost risk‐mitigation strategy implementable within existing clinical infrastructure.

Regarding dietary and nutritional counseling, clinicians should routinely obtain dietary and supplement history in all patients with known or suspected LQTS and in those receiving QT‐prolonging medications. Patients should be counseled to avoid concurrent consumption of grapefruit juice, licorice‐containing products, energy drinks, and herbal or over‐the‐counter preparations with documented QT‐prolonging or CYP‐inhibitory properties, particularly in the context of co‐administered QT‐active drugs or genetically determined repolarization vulnerability [28]. Where pharmacogenomic testing is available, identification of CYP3A4/3A5 poor‐metabolizer status should trigger heightened vigilance regarding dietary CYP inhibitor exposure.

## 9 Conclusion

The biology of QT prolongation traces an arc from the atomic‐scale interactions of ion channel proteins with their pharmacological ligands and genetic variants, through the metabolic and pharmacological forces that amplify or modify repolarization reserve in the general population, to the population‐scale detection of arrhythmic risk by deep learning algorithms trained on millions of electrocardiograms. This review has traced that arc across five domains—molecular pathogenesis, acquired risk, clinical stratification, therapeutic management, and artificial intelligence—and the coherence of the picture that emerges justifies an integrative rather than domain‐specific analytical frame.

Several conclusions stand out with reasonable evidential support. First, the molecular substrate of QT prolongation is more heterogeneous than the three‐gene LQTS framework suggests, and variants with dual electrophysiological consequences, post‐translational regulatory defects, and novel gene involvement are increasingly being identified through comprehensive genetic testing and functional characterization. Second, the clinical risk of QT prolongation is not adequately captured by QTc thresholds alone—T‐wave morphology, genotype, sex, symptomatic history, and dynamic treatment response all contribute independent prognostic information, and risk stratification must be applied longitudinally rather than once at diagnosis. The validity of the QTc measurement itself deserves renewed scrutiny: formula choice introduces systematic measurement bias that propagates through all downstream risk calculations and AI model outcomes. Third, metabolic risk factors—specifically insulin resistance, nonalcoholic fatty liver disease, and regional adiposity—constitute an underappreciated, independently acting tier of QT risk; their systematic exclusion from current risk instruments represents a structural gap with real‐world consequences. These associations are observationally well‐established but remain to be confirmed in prospective interventional designs before formal guideline incorporation is warranted. Fourth, nutritional factors—grapefruit juice, licorice, energy drinks, and over‐the‐counter supplements—interact with genetic susceptibility and prescribed pharmacotherapy through pharmacokinetic, electrolyte‐mediated, and gene–nutrient interaction pathways that are clinically actionable now, through dietary counseling and pharmacogenomic‐informed prescribing.

Fifth, genotype‐targeted pharmacotherapy—particularly mexiletine in LQT3 and emerging evidence in LQT2—offers a compelling alternative to escalating device therapy, and LCSD extends this pharmacological‐to‐surgical escalation ladder. ICD therapy, while irreplaceable in high‐risk patients, is being applied with patterns that likely include significant overimplantation. Sixth, the nonpenetrant LQTS phenotype—genotype‐positive but without any electrocardiographic expression of disease—carries near‐population‐level arrhythmic risk, a finding that substantially refines the management calculus for genotype‐positive family members identified through cascade screening. Seventh—and perhaps most consequentially for the near‐term future of the field—artificial intelligence–based risk prediction models substantially outperform current clinical risk instruments for both drug‐induced QT prolongation and sudden cardiac death in retrospective cohort benchmarking studies. Deep neural network analysis can now distinguish congenital from acquired QT prolongation at the ECG level, addressing a long‐standing diagnostic challenge. However, their clinical implementation is conditional on prospective effectiveness data demonstrating that model outputs change decisions and improve outcomes—data that have not yet been generated for any currently available platform—as well as on external validation in demographically diverse populations and on interpretability frameworks that allow clinicians to understand and appropriately trust model predictions.

The path from molecules to machines in this domain is not a replacement of biological understanding by algorithmic prediction—it is an integration of both. The cardiologist who understands why a KCNH2 pore‐region mutation carries three times the arrhythmic event risk of a non‐pore variant, who recognizes NAFLD and insulin resistance as independent electrophysiological risk modifiers, who counsels patients on grapefruit juice and energy drinks with the same evidence‐based rigor brought to drug prescribing, and who applies a formula‐appropriate QTc correction to a population with known heart rate distribution characteristics is better positioned to interpret an AI model's risk prediction than one who treats the model output as a black‐box oracle. Building that integrated framework—across molecular genetics, clinical cardiology, metabolic medicine, pharmacology, and computational science—is the central task before the field.

## Author Contributions

**Mojtaba Farjam:** conceptualization, methodology, supervision, funding acquisition, writing – review and editing. **Mohammad Hosein Yazdanpanah:** writing – original draft preparation, formal analysis, writing – review and editing. **Narges Fereydouni:** conceptualization, writing – review and editing, project administration.

## Conflicts of Interest

The authors declare no conflicts of interest.

## Data Availability

No new datasets were generated or analyzed in this review. All data discussed are available in the primary publications cited herein.
